# Football, Culture, Skill Development and Sport Coaching: Extending Ecological Approaches in Athlete Development Using the Skilled Intentionality Framework

**DOI:** 10.3389/fpsyg.2021.635420

**Published:** 2021-07-08

**Authors:** James Vaughan, Clifford J. Mallett, Paul Potrac, Maurici A. López-Felip, Keith Davids

**Affiliations:** ^1^School of Human Movement and Nutritional Sciences, The University of Queensland, Brisbane, QLD, Australia; ^2^Research and Development Department, AIK Football, Stockholm, Sweden; ^3^Faculty of Sport and Health Sciences, Technical University of Munich, Munich, Germany; ^4^Department of Sport, Exercise and Rehabilitation, Northumbria University, Newcastle upon Tyne, United Kingdom; ^5^School of Public Health, Physiotherapy and Sports Science, University College Dublin, Dublin, Ireland; ^6^Department of Psychological Sciences, Center for the Ecological Study of Perception and Action, University of Connecticut, Storrs, CT, United States; ^7^Team Sports Department, Futbol Club Barcelona, Barça Innovation Hub, Sant Joan Despí, Spain; ^8^Skill Acquisition Research Theme, Centre for Sports Engineering Research, Sheffield Hallam University, Sheffield, United Kingdom

**Keywords:** ecological dynamics, sociocultural constraints, creativity, skilled intentionality, football, coaching, value-directedness, ecological psychology

## Abstract

In this manuscript, we extend ecological approaches and suggest ideas for enhancing athlete development by utilizing the Skilled Intentionality Framework. A broad aim is to illustrate the extent to which social, cultural and historical aspects of life are embodied in the *way football is played* and the *skills young footballers develop* during learning. Here, we contend that certain aspects of the world (i.e., environmental properties) are “weighted” with social and cultural significance, “standing out” to be more readily perceived and simultaneously acted upon when playing football. To comprehend how patterns of team coordination and athletic skill embody aspects of culture and context we outline the value-directedness of player-environment intentionality. We demonstrate that the values an individual can express are *constrained* by the character of the social institutions (i.e., football clubs) and the social order (i.e., form of life) in which people live. In particular, we illuminate the extent to which value-directedness can act as a constraint on the skill development of football players “for good or ill.” We achieve this goal by outlining key ecological and relational concepts that help illustrate the extent to which *affordances* are value-realizing and intentionality is value-directed (exemplified, by footballers performing in a rondo). To enhance coaching practice, we offer: (a) insights into markers of skilled intentionality, and (b), the language of skilled intentions, as well as highlighting (c), an additional principle of Non-linear Pedagogy: *Shaping skilled intentions*, or more precisely shaping the value-directedness of player-environment intentionality. We contend that, if sport practitioners do not skilfully attend to sociocultural constraints and shape the intentions of players within training environments and games, the social, cultural, and historic constraints of their environment will do so: constantly soliciting some affordances over others and directing skill development.

## Introduction

Played in over 200 countries around the world, football embodies aspects of the society, and culture in which it is embedded. In particular, distinct patterns of team coordination and exceptional athletic skill have been seen to embody aspects of culture and context (Araújo and Davids, 2016; Uehara et al., 2018). This is best exemplified by the deceptive dribbling skills of renowned Brazilian football players, with the “way they play” and the “skills they display” embodying celebrated aspects of Brazil’s cultural identity (Uehara et al., 2016, 2018).

Since the inaugural World Cup in 1930, the relationship between culture and skilled performance has been observed with interest. However, this relationship has not inspired sustained academic attention, and the relations between sociocultural aspects of life and athlete development practices have remained relatively unexplored (Uehara et al., 2016; Rothwell et al., 2018, 2019). We attribute this, in part, to limitations of dominant theoretical and conceptual frameworks underpinning sport science, psychology and coaching (Davids and Araújo, 2010; Araújo and Davids, 2011); specifically, the traditional psychological dichotomies of perception-action, organism-environment, subjective-objective, and mind-body (Lobo et al., 2018).

The extent to which sport science and coaching practice have been adversely affected by western dualisms (Baggini, 2018) and traditional psychological dichotomies was exposed by Davids and Araújo (2010). Burdened by the assumption of an organism-environment dichotomy, a significant body of research applied to sport is narrowly focused on the individual athlete (Henriksen and Stambulova, 2017). This has created an organismic asymmetry, described as an inherent bias toward seeking behavioral explanations within the boundaries of the individual athlete (i.e., organism) (Araújo and Davids, 2011). Dichotomies and dualisms continue to plague sport research and applied practice. For example, football is analyzed and explained by assuming an attack-defend dualism, whereby the game is viewed from the separate perspectives of either the attack or defense (López-Felip, 2019; López-Felip et al., 2020). This fragmented perspective obscures and ignores the dynamic relations and constant co-adaptation between attack and defense (Passos et al., 2016; López-Felip and Turvey, 2017). This recognition has led scholars to recommend that the specific ontology of sporting activity be reconsidered (see López-Felip, 2019; López-Felip et al., 2020).

Limited by dichotomies and dualisms at multiple levels, a majority of coach education, as well as coach practice, remains unable to account for the deeply contextualized and ecological relations at the heart of sport and life (Bowes and Jones, 2006; López-Felip and Turvey, 2017). In this paper, we highlight the opportunity to recontextualize athlete development and coaching practice by identifying how to harness social, cultural, and historic aspects of life using ecological approaches (Rothwell et al., 2018; O’ Sullivan et al., 2021).

## Prologue: the Need To Recontextualize Our Ways of Knowing in Football

Traditional psychological dichotomies and an “organismic asymmetry” (Dunwoody, 2006) have de-contextualized and de-humanized our ways of knowing, our methods of working and the practices applied in sport and education (Bocchi et al., 2014; Vaughan et al., 2019). van der Kamp et al. (2019) explained that the content of most education, as well as the methods used to educate, focus almost exclusively on communicating abstract and decontextualized knowledge *about* the world. However, decontextualized knowledge and associated methods of coaching cannot account for the contextual complexity, ecological relations and incalculable system semantics of team sports (Bowes and Jones, 2006; López-Felip and Turvey, 2017; Vaughan et al., 2019). Therefore the need to re-contextualize and re-humanize sports coaching and educational practices is apparent (Jones et al., 2010). Taking a step in this direction this article argues that knowledge *of* the performance environment is better suited for supporting perceptual learning, intentionality, and the split-second adaptations of players during the continuous interactions of team sport (Chow et al., 2020).

Currently, football coaches and players are educated using a smorgasbord of abstract and decontextualized *football concepts*, i.e., switches of play, defensive triangles, compact lines, inner corridors, third man runs, overlaps, width utilization, etc., While these concepts might aid conversations between coaches, we argue that communicating second-hand knowledge *about* football to players is largely ineffectual if one aims to optimize skill development and team coordination. Skill development is the term we use to encompass skilled performance, perceptual learning, and collective behavior in sport (Araújo et al., 2017; López-Felip, 2019; Woods et al., 2020b). These related processes are reliant on players becoming directly attuned to, and adapting with, the dynamic properties *of* football performance environments.

*Knowledge of* a performance environment helps each athlete (i.e., organism) to perceive the surrounding layout of their performance environment at the scale of their body and action capabilities (Araújo and Davids, 2009). James Gibson (1966) proposed that individuals use this type of knowledge to regulate behaviors during continuous interactions with objects, events, other people, surfaces and features of the terrain in the environment. To exemplify, knowledge *of* the performance environment may be comprised of surrounding information from the continuous movements of opponents, teammates and the ball in football, revealing available affordances of gaps, obstacles, locations and spaces for achieving competitive aims. Nevertheless, *talking about* football concepts that are abstract and decoupled from the environmental properties *of* the performance environment, remains the predominant approach of much coach education and coaching practice.

Due to the epistemological insufficiencies (dualisms, dichotomies and decontextualization) of coach education, the “toolboxes” of football coaches often contain an ensemble of isolated and unrelated coaching methods and football concepts. This situation is similar to a collection of unrelated puzzle pieces that don’t seem to connect and align and therefore do not provide a unified picture of player development (Vaughan et al., 2019). The tendency to copy and paste concepts or practices without sensitivity to the social, cultural or historic context (Stambulova and Ryba, 2014) might explain how coach education amasses collections of disparate puzzle pieces with little, or no, relation to one another. However, this does not explain why these concepts are decoupled from, and unrelated to, key environmental properties in football.

Bocchi et al. (2014) offer insight into the situation by highlighting a historic, and path dependent^1^, overreliance on reductive analysis. They stated that in the attempt to generate clear and transmittable knowledge, positivism did not engage with the social, cultural, and contextual interdependencies of human life. Baggini (2018) has argued that the dominance of this worldview has consigned relational concepts to the historical texts of east-Asian philosophies and the walls of boutique yoga studios. In football, limited ways of knowing amplify the reductive, overly analytical and decontextualized approaches to coach education and on field coaching practice.

### The Fragmented Picture of Player Development in Football

The continual reduction and fragmentation of disciplines, knowledge, and human experience in sport has led to the growing recognition that a positivist hegemony is limiting our understanding of human behavior (Uehara et al., 2016; Balagué et al., 2017). Overly reductionist and analytical methodologies create isolated fragments of decontextualized knowledge (Alhadeff-Jones, 2009). These fragments are unable to account for the sociocultural complexity and sub-system interrelations of athletes and environments and consequently present deconstructed and unrelatable picture of athlete development (Balagué et al., 2017; Rothwell et al., 2020). In applied settings, the reductive methods of disciplinary specialists tend to have create silos of practice. For example, strength and conditioning coaches, medical departments, nutritionists, psychologists and sports coaches hold information on important “pieces of the athlete development puzzle.” However, these pieces are often decontextualized and ignore the holistic nature of performance and development and are founded on incompatible ontological and epistemological perspectives. In professional football, decontextualized knowledge *about* the environment and statistical models dominate the work of coaches and practitioners. In particular, quantitative data tends to dominate the assessment and planning of training sessions, leaving considerations of skill development and motivation largely ignored in favor of counting the “frequency of ball contacts” (as a surrogate for skill acquisition), number of “sprints made” and “kilometers run” (as surrogates for conditioning) (Vaughan et al., 2019).

Aligned with rationalistic and linear assumptions, the hegemony of reductive methods has had serious repercussions for coach education in football (Bowes and Jones, 2006; Vaughan et al., 2019). Traditional methodologies remain focused on knowledge *about* the game, with football concepts used to prescribe a sequence of “best decisions” and “optimal” solutions aligned to predetermined game models or tactical plans. However, as Araújo et al. (2017) have clarified, there can be no optimal chain of “best decisions” in team sports like football. The interdependent, relational and situational game dynamics mean that the “most functional decision at any moment may compromise future decisions” (p. 5). As López-Felip and Turvey (2017) explained, the interactions of football players are deeply contextualized and entirely dependent on emergent information of *when*, *where*, and *who*.

No amount of abstract representations, football concepts or prescriptive coaching plans are able to account for the reality that the decisive interactions of players (e.g., passes, dribbles, tackles, interceptions, fakes, and feints) are emergent and unpredictable and unknowable (Araújo et al., 2017; Denison et al., 2019). Therefore, and as Bowes and Jones (2006) explained, the process of coaching defies explanation by the rationalistic and linear assumptions on which such work is currently based. Despite this limitation, traditional football concepts focused on knowledge *about* the game dominate the content of player development plans and the curriculum of coach education. In practice, these concepts have become the “learning outcomes” and topics of training sessions and remain coupled to methods that encourage the verbal recall of abstract concepts. At the end of a training session a coach might ask, *what did we work on today?* If a player is able to accurately recall the verbal explanations that took place during the training session, it is often taken as proof of learning. Even if that player had not displayed any of what they talked about during the training session itself.

### Recontextualizing Football and Coaching Practice

The aim of this paper is to illuminate relational concepts and propose initial steps to recontextualize coaching and athlete development practices. By combining Ecological Dynamics (Araújo et al., 2006, 2017) and the Skilled Intentionality Framework (SIF: van Dijk and Rietveld, 2017; Rietveld et al., 2018) we outline the value-directedness of player-environment intentionality. Specifically, we illustrate the extent to which social, cultural, and historic aspects of life influence the skill development of footballers. Extending ecological approaches allows us to overcome a history of dichotomies and dualisms that currently limit the theoretical and conceptual frameworks underpinning sport science, psychology and coaching (Davids and Araújo, 2010; Araújo and Davids, 2011).

To achieve this aim, we begin by discussing Ecological Psychology as a framework to dissolve the dualisms, dichotomies, and decontextualization that plagues sport research and applied practice. Founded in the Gibsonian branch of Ecological Psychology (Gibson, 1979; Araújo, 2009), we conceive the relation between values and direct perception through the work of Hodges and Baron (1992), who contend that values act as constraints on affordance utilization (fully introduced later). Our argument focuses on an aspect of intentionality Hodges and Baron (1992) called value-directedness, and we attempt to illustrate the extent to which intentionality is value-directed and affordances are value-realizing. Connecting to, and extending the literature on Ecological Dynamics, we illustrate how aspects of society and culture co-create a value-directedness (of intentionality) that acts as a *constraint* on the use of information to regulate actions, affordance utilization, and skill development. To aid sports practitioners in understanding and adapting to this variety of sociocultural constraint, we offer an additional principle to non-linear pedagogy that encompasses constraint-led coaching (Chow et al., 2016; Renshaw and Chow, 2019). Namely, the need to shape the value-directedness of player-environment intentionality, or more concisely, the need to continuously shape skilled intentions during performance interactions.

## Ecological Approaches

To conceptually illustrate athletes *relations* with environments and theoretically discuss their implications for skill development we carefully extend key concepts founded in the Ecological Psychology of J. J. Gibson (1904–1979) and E. J. Gibson (1910–2002) (Gibson, 1979, 1988). Ecological Psychology offers a way of moving beyond the traditional psychological dichotomies (entrenched in cognitivism and behaviorism) to understand perception, cognition, action and perceptual learning from an ecological perspective (Lobo et al., 2018). In this paper we, extend key Gibsonian concepts, and apply them to athlete development, by combining the theoretical and conceptual frameworks of Ecological Dynamics (Araújo et al., 2006, 2017) and the SIF (van Dijk and Rietveld, 2017; Rietveld et al., 2018). In particular we follow the reasoning of Rietveld et al. (2018) and aim to demonstrate the extent to which:

Affordances always have to be understood in the context of an ecological niche that implies the form of life of a certain kind of animal. Therefore, we define an affordance as a relation between (a) an aspect of the (sociomaterial) environment and (b) an ability available in a “form of life” [Wittgenstein, 1953, cited in Rietveld et al. (2018)] (p.5).

We contend that non-reductive ecological approaches to understanding the underpinning role of the perception-action relationship provides a foundation for greater synthesis in sport. In particular, Fajen et al. (2009) argued that ecological accounts can lead toward a unified framework for sports that is theory-driven and practice-oriented (López-Felip and Turvey, 2017). By fostering such synthesis, ecological approaches might unite the art of coaching with the science of sport.

### Ecological Dynamics and the Skilled Intentionality Framework

Ecological Dynamics underscores how an athletes relations to, and interdependence with, an environment facilitates specific behaviors in sport, emphasizing the importance of interactions that are continuously regulated by information from the performance environment (Araújo and Davids, 2016; Teques et al., 2017). It has been known for some time that, as individuals enrich their skills, they become better attuned to the informational properties of the performance environment which specify their actions. For example, Runeson et al. (2000) demonstrated that, while perceivers may not initially utilize specifying information to successfully regulate their actions, with learning and experience they learn to do so, resulting in more successful levels of task performance (see Araújo and Davids, 2009; Renshaw et al., 2016 for applications in sport). Gibson (1966) proposed that each individual’s central nervous system resonates to surrounding information available in energy sources. For him, resonance is not some magical entity like an abstract representation encoded in the brain, but rather a central process in explaining *how* perception of the environment emerges through continuous interactive behaviors of an organism. Rather like an antenna, Gibson (1966) proposed that learning and experience resulted in more refined and precise resonance or attunement to surrounding information, available in surrounding energy arrays, which specifies actions (for further explanation see Teques et al., 2017). The concept of resonance is more than a metaphor and can explain how, through learning and experience, neurobiological systems can resonate to the invariant properties of a surrounding energy array, attuning an individual to information from a performance environment (Gibson, 1966; Raja, 2019). These ideas from ecological psychology imply that, in sport, resonance may play a role in helping athletes enhance their functional interactions with their performance environment through becoming better attuned (e.g., synchronized) to the patterns of structured surrounding energy (information), acquired and developed through learning to actively perceive and utilize available affordances of the performance environment.

This description of ecological dynamics captures how the athlete-environment system becomes highly interconnected, attuned, and resonant, making it problematic to practice performance behaviors in isolation, away from typical contexts. Indeed, behavior and skill development can only be understood in accordance with both, the characteristics of a performer and the characteristics of a performance environment simultaneously (Araújo et al., 2017). Therefore, the process of skill acquisition is better conceptualized as skill adaptation or development: the deep attunement to, and resonance with, the surrounding information in a practice or performance environment (Button et al., 2020; Chow et al., 2020).

In football, skill develops as players become perceptually attuned to opportunities for action (affordances) presented by properties of the playing environment, e.g., the ball, other players, field markings, playing surface, and weather (Coutinho et al., 2016). However, not all environmental properties are equal and balanced. Certain aspects are seemingly “weighted” with differential social and cultural significance: soliciting attention and “standing out” to be more readily perceived by actors (van Dijk and Rietveld, 2017). For example, the gap between an opponents’ legs resonates with cultural significance for Brazilian footballers. Presenting an alluring invitation to deceive opponents (with a Panna/tunnel/nutmeg) and embody a style of play (Ginga) consistent with Brazil’s cultural identity^2^ (Uehara et al., 2018). Such examples capture the dynamics of affordance solicitations (Withagen et al., 2017) in sport and underscore the need to appreciate how sociocultural-historic constraints frame perception-action (Hodges and Baron, 1992).

Within Ecological Dynamics, the interactions of teams and players are deeply interconnected and continuously shaped by the *constraints* of environments and tasks. Constraints act as information and are related to each individual, task and environment, they are also embedded in sociocultural contexts and shaped by sociocultural forces (Vaughan et al., 2019). By continually shaping the self-organizing tendencies of performers and teams’ constraints continuously exert an influence on performance and learning (Davids et al., 1994). In sport, sociocultural constraints are the dynamic principles that shape the self-organizing patterns of group dynamics in performance contexts (Araújo and Davids, 2016). In order to illustrate social and cultural processes as key constraints on skill development we follow the recommendation of Araújo et al. (2017) and focus on contexts and relations, and particularly on affordances (Rietveld et al., 2018).

### Perception Is of Affordances

Central to Ecological Dynamics, the SIF, and at the core of the athlete-environment system, is Gibson’s (1979) theory of affordances. In Ecological Dynamics perception is of affordances and behavior is self-organized under constraints. In contrast to traditional perspectives, behavior is not imposed from *inside* (i.e., the mind) or *outside* (e.g., verbal instructions) (Araújo et al., 2017)^3^. Hodges and Baron (1992) contended that it was Gibson’s refusal to accept that behavior is imposed from inside or outside that led him to reject the dualisms and value-free science of western positivism. Gibson started to dissolve the dichotomies of traditional psychology by creating the ecological account of perception-action and the novel ontology of affordances:

An affordance is neither an objective property nor a subjective property; or it is both if you like. An affordance cuts across the dichotomy of subjective-objective and helps us to understand its inadequacy. It is equally a fact of the environment and a fact of behavior. It is both physical and psychical, yet neither. An affordance points both ways, to the environment and to the observer (Gibson, 1979, p. 129).

Addressing the keystone dualism between organism and environment affordances were described as the embodiment of the value offered by the organism-environment ecosystem “for good or ill” (Gibson, 1979, p. 127). As such affordances “avoid both the false reduction of the social reality to merely physical and the equally false reduction of biological reality to the cultural” (Reed, 1988, p. 310). For example, the edge of a cliff top can offer opportunities/affordances for climbing over and/or locomoting along (i.e., walking, running, rolling on a bike). Simultaneously, it also offers the opportunity to pose for photographs (to be posted in online communities) in bracing weather and/or beautiful surrounds, it also provides possibilities for falling off.

Building on Gibson’s seminal work the SIF clarifies how complementary insights on affordance responsiveness from philosophy, ecological psychology, emotion psychology and neurodynamics hang together in an intertwined way (Rietveld et al., 2018). Central to the SIF is the notion of *sociomaterial entanglement*, which stresses that affordances are not just situated in the materiality of the immediate behavioral setting, but entwined within a more culturally encompassing, socially shared and historically developed constellation of practices and forms of life (van Dijk and Rietveld, 2017). Guided by the SIF we aim to outline the world’s *constitutive sociomaterial entanglement* with examples of affordance utilization in football.

### Nested Affordances and Skilled Intentionality

Turvey (1990) stated that affordances and effectivities can only be discovered and used when intentionality and lawfully specified possibilities are coordinated. These two aspects, the intentionality of an agent and the aptness of the situation, give rise to the relational properties of effectivity and affordance. The first, effectivity, describes the property of the agent which allows him or her to utilize a feature of the environment. Affordance, on the other hand, describes the property of the environment which the agent orients to and utilizes. More formally:

Situation X *affords* activity Y for organism Z on occasion O if and only if X and Z are mutually compatible on dimensions of relevance to Y.
Organism Z *effects* activity Y in situation X on occasion O if and only if Z and X are mutually compatible on dimensions of relevance to Y (Turvey et al., 1981; López-Felip and Turvey, 2017).

“Lawfully specified” is important for ensuring that the perception of affordances is *direct*, that is, the agent does not need a representation of (or knowledge *about*) an affordance, or have to *think* to utilize it, they simply perceive it (Turvey et al., 1981). These possibilities are created through physical laws, material substances and properties of the environment. For example, a skilled football player perceives an affordance to score a goal when properties of the visual field lawfully specify openings in the defense (in the direction of the goal), and he or she has, through training, developed the effectivity to kick the ball accurately through the opening.

However, an affordance to score is not perceived without the intention to score, which is embedded within the social practice of “scoring a goal.” Both of which are culturally embedded in the sociocultural practice and form of life of football (van Dijk and Rietveld, 2017). Adjacent pillars/trees and lifeless jumpers (upper body clothing) can be transformed into goal posts and afford numerous opportunities for action once they are intended to be used as goals.

To exemplify the sociomaterial entanglement of affordances, imagine that I am in a public park, kicking a football back and forth (passing the ball) with my teenage niece. When I move our jumpers to create a goal (material space) and gesture with my hands (social coordination) – mimicking the position of a goalkeeper in football – I am simultaneously changing the material space and reconfiguring the sociomaterial environment. This alters the relevant field of affordances, which fluctuate from passing opportunities (two people with a ball) to shooting, scoring and saving opportunities (afforded by two people, a ball and a goal). The manipulation of space, alongside the inclusion and placement of goals (two key task constraints), exemplify the type of constraint manipulation that is a central pedagogical principle of constraints-based coaching within Non-linear Pedagogy and Ecological Dynamics (Renshaw et al., 2019).

Affordances are also subject to, and unfold in interaction with, changes in the surrounding sociomaterial environment, inclusive of people (van Dijk and Rietveld, 2017). Imagine that my niece is unfolding a (seemingly) powerful shot, when during her downswing she notices a father and child walking behind our “jumpers for goal posts.” In a split-second her affordance for a powerful shot dissolves (in the social milieu) and she unexpectedly and skilfully aborts (i.e., fakes) the shot. In this moment she demonstrates skilled intentionality, defined as a “skillful responsiveness to multiple nesting and nested affordances simultaneously” (van Dijk and Rietveld, 2017 p. 9) “for good or ill” (Gibson, 1979, p. 127) within our overlapping forms of life^4^ (i.e., humans in public parks and football players).

### Skilled Intentionality: The Foundation of Creativity in Football

In the moment my niece perceives and acts (a simultaneously cycle) on her affordance to shoot, attuned to a space in the goal to my right. I pre-reflectively move toward the unfolding affordance to save the “would be” shot down low to my right (a type of shared affordance, for details see Araújo and Davids, 2016). However, as she aborts (i.e., fakes) the shot I cannot fully abort the (would be) save and my inertia carries me off balance as I attempt to change direction. My niece compounds my embarrassment by cheekily (and safely for father and child walking behind our jumpers for goalposts) rolling the ball into the open goal to my left, leaving me sprawled on the ground.

Here we contend that footballers embody (i.e., display) skilled intentions when they deceive and unbalance opponents with fakes and feints (introduced later as “markers” of skilled intentionality in football). Exemplified by a goal (seen here^5^, Bundesliga, 2013) that was recently described as the most spectacular goal in the history of German football by Jürgen Klopp (Aarons, 2020). In football, coaches have described creativity as “the art of deception” (Vaughan, 2014, p. 93), with affordances to deceive opposition players nested (hidden) within the relevant affordances that “stand out.”

### Nested, Deceptive, Multiple, Simultaneous and Shared Affordances

Adopting an ecological perspective implies that, in football, the skilled intentionality I can display is, in part, reliant upon the intentions of my teammates, and the opponents around me. Specifically, we suggest that skilled intentionality is dependent upon the shared perception of nested affordances within unfolding interactions between attackers and defenders on the football pitch. To give one brief example, consider the extent to which an opponent’s affordance to intercept my pass is related to, and nested within, our shared perception of the passing opportunity/affordance itself (Araújo and Davids, 2016). Equally, an affordance to fake a pass, and deceive my opponent, is reliant upon that player perceiving both, the opportunity for me to pass, and simultaneously their (nested) invitation to intercept my pass. In other words, I can only successfully fake or feint a pass, shot, or dribble, if my immediate opponent’s skilled intentionality (perceptual skill/attunement) allows them to perceive my action opportunities. For this reason, skilled intentionality is construed to take place within, and is inextricably related to a form of life, and the relevant field of affordances that stand out within a sociocultural practice, like football (van Dijk and Rietveld, 2017). As such, we contend that skill development and creativity in football is inextricably reliant on the perception of shared affordances and therefore the skilled intentionality of footballing forms of life (Araújo and Davids, 2016; Vaughan et al., 2019). Footballing forms of life may be equated to the regional and national playing styles that can be characterized as sociocultural artifacts (Rossing and Skrubbeltrang, 2016). Rietveld et al. (2018) point out that humans develop skills within an existing form of life, which leads us to contend that football players develop skills related to the relevant affordances that stand out in their footballing form of life. Historically, the development of skill sets coupled to a distinct style of play has been exemplified by players (e.g., Andreas Iniesta and Xavi Hernandez) and teams at FC Barcelona (Vaughan et al., 2019).

### Constitutive Relationships: Two Sides of the Same Coin

van Dijk and Rietveld (2017) describe the constitutional entanglement of sociocultural practices (e.g., football) and affordances (e.g., opportunities to pass) by situating them as “two sides of the same coin” (p. 2):

It is an example of a constitutive relation because (i) the practice [e.g., football] and the affordances that take shape within it [e.g., opportunities to pass] are interdependent: any affordance will imply a practice for realizing it and any practice [e.g., football] will imply a landscape of available affordances [e.g., movement opportunities in football]. Furthermore (ii) practices and affordances do not admit of a prioritization (p. 4, text in brackets added).

We extend this interdependence to relevant (fields of) affordances in footballing forms of life and the skills footballers develop over the weeks, months, and years of athlete development. Meaning that the skills athletes develop are reliant upon the affordances that make up their practice environments. Therefore, affordances and skills are also two sides of the same coin (which do not admit a prioritization) and athlete development is deeply ecological. More broadly, however, the ontological notion of *constitutive sociomaterial entanglement* proposed in the SIF sees forms of life, sociocultural practices, relevant fields of affordances and skills as constitutive relations and aspects of the same whole that continuously form each other (van Dijk and Rietveld, 2017). This expanded view of intentionality captures social and cultural aspects while recognizing that “intentionality characterizes the system, not just biological organisms within the system” (Hodges and Baron, 1992, p. 270). These deeply relational concepts are not fertile in western worldviews, so to exemplify our conception of intentionality we introduce the non-dualistic notion of *Yinyang:*

That something [e.g., intentionality] can be yin [i.e., player] and yang [i.e., environment] at the same time is not paradoxical. The same is true of any relational property something can be to the left of one thing, to the right of another… Yinyang [i.e., intentionality] shares this dependence on context because it is inherently concerned with relationships… the main focus of Yinyang [i.e., intentionality] is not to describe the world… rather it enables us to live well in it (Baggini, 2018, p. 238 text in brackets added).

Akin to Yinyang, intentionality is entirely dependent upon context, inherently concerned with relationships (e.g., affordances), and may help us to live well, and move skilfully through the world. Having outlined key ecological and relational concepts we now aim to illustrate the extent to which affordances are value-realizing and intentionality is value-directed (Hodges and Baron, 1992).

## Intentionality and Value-Directedness

Interviewed at the end of his career, Gibson said: “I have been moving toward a psychology of values instead of a psychology of stimulus” [Locker, 1980, as cited in Reed (1988), p. 296]. Within the framework of Gibson’s affordances, Hodges and Baron (1992) explained that a car stopping for a child illustrates a value-realizing relation, and that “such a relation is not arbitrary in the way in which stopping for the red light is [social rule], but it is not inviolable in the same way being stopped by the brick wall would be [physical law]” (p. 271, text in brackets added). For Hodges and Baron (1992) “perceiving is a value-realizing activity and… the constitution and detection of affordances is a partial realization of values” (p. 263).

To exemplify the value-realization of affordances Hodges and Baron (1992) described that:

Cups with liquids in them share a dual set of affordances: drinking and spilling. It is no accident of culture that cups for babies are designed to make drinking affordances easier for the child to realize than spilling affordances…Wise cultures physically structure and socially guide children’s activities to make “right ways” easier to realize than “wasteful,” wanton ways (p. 273; footnote added).

Hodges and Baron (1992) point out that even the most fail-safe baby cup affords spilling, but in their design, they clearly privilege drinking. This culturally resonant and relevant (i.e., “standing out” in a tactic and implicit way) field of affordances infers that the liquid is a resource of value and significance. The design of the cup and the sociocultural practice of drinking contribute toward the value realization embodied in the affordance to drink. However, Hodges and Baron (1992) also note that in *affluent cultures* whereby children are presented with surplus food and drink the value embodied within the drinking affordance is less likely to be directly perceived and realized (Hodges and Baron, 1992). Adopting these perspectives alongside the SIF, we aim to reveal the extent to which (relevant fields of) affordances “stand out” with sociocultural significance: Constituting and inviting the partial realization of social and cultural values in a context-sensitive and embodied way.

### The Rondo (i.e., Square, Box): Values Realized in Passing Affordances

In the same way that the culturally embedded design of baby cups infers that the liquid is a resource of value and meaning, the design of a rondo illuminates ball possession as a resource of value in football. A rondo is a training exercise design that encourages a greater number of “in possession players” (e.g., three, four, five etc.) to keep possession of the ball against a lesser number of “out of possession players” (e.g., one, two, three) who attempt to take (intercept or tackle) the ball from the opponents. The rondo spotlights passing and receiving opportunities by creating a relevant field of affordances that invite players to embody (i.e., partially realize) the value in teamwork and collaboration. In other words, players must coordinate their movements to create passing affordances and maintain possession. However, while the isolated design (pen on paper) of a rondo encourages players to detect passing affordances, a rondo does not exist in a sociocultural vacuum, and the cultural context may distort the value-realization of passing affordances (in the same way that affluent cultures might distort the value embodied in drinking affordances).

Rasmussen et al. (2017) explained, “players’ intentions may vary between showing off, orchestrating the game, or contending against peers” (p. 9) and that having purpose with one’s actions is a category of intentionality. Crucially, however, Hodges and Baron (1992) remind us that intentions and intentionality are not merely the realm of the individual:

Intentionality characterizes the system, not just biological organisms within the system. Thus, intentionality in the sense of value-directedness characterizes environmental structures [i.e., footballs form of life] and processes [i.e., football rondo], as much as it does the organisms [i.e., players] who shape and are shaped [e.g., skill development] by those structures and processes. This implies that *values are necessary constraints on both the constitution and the selection of affordances* (p. 270; text in brackets and italics added).

Consider the situation whereby I am passing to a teammate with whom I am competing for a contract, or even a place in the weekends starting XI. Does the value-directedness of this situation shape passing affordances to covertly sabotage my teammate? Does the player-environment intentionality I experience invite me to strike the ball a fraction too hard or bobble the pass, making it difficult for my teammate to control? Our argument now focuses on the aspect of intentionality that Hodges and Baron (1992) called value-directedness, and we continue to illustrate the extent to which intentionality can be considered value-directed in football.

## Player-Environment Intentionality in Football

In football, intentionality relates to a players’ interactions with the performance environment being directed at *something* or *someone* (Rasmussen et al., 2017). In this way intentionality fosters a broad orientation toward the environment (i.e., utilization of available affordances) and is best conceptualized and experienced as a “directedness” (van Dijk and Rietveld, 2017). Intentionality is not about explicit goals, tactics or techniques that need be applied. It is broader, more flexible and adaptive to multiple action possibilities. Explaining the directedness of player-environment intentionality, Rasmussen et al. (2017) suggested that “players direct their actions toward certain aspects of the world, but their intentional state [i.e., value-directedness] determines what kind of aspects are targeted and which affordances are discovered, exploited, and invented” (p. 9, text in brackets added).

### Value-Directedness as a Sociocultural Constraint

In situations whereby value-directedness is experienced as rigid and inflexible we contend that it will limit skill development opportunities (affordance detection and selection), with some affordances “standing out” and nested affordances undiscovered.

As previously noted by Rasmussen et al. (2017), players’ intentions can vary between showing off, orchestrating the game, or competing against peers and teammates. We propose that these contemporary intentions manifest in concert with three cultural (i.e., form of life) changes identified in America, Canada and Britain over the last 30 years, specifically: “(a) the emergence of neoliberalism and competitive individualism, (b) the rise of the doctrine of meritocracy, and (c), increasingly anxious and controlling parental practices” (Curran and Hill, 2019, p. 3). As such, players’ intentions are value-directed by, and resonate with, forms of life that prioritize: neo-liberal competition, individualism, constant comparison, evaluation and social status (social media “likes” and “follows”) (Roderick, 2006; Potrac et al., 2013; Vaughan, 2020).

Here, we contend that the social and cultural (i.e., form of life) changes identified by Curran and Hill (2019) might privilege a value directedness that emphasizes interpersonal competition to such an extent that affordances to truly collaborate can be largely overshadowed in football and sporting environments subject to neoliberal ideology and rampant commodification (exemplified in the previous rondo example and these papers, Potrac et al., 2013, 2017; Gale et al., 2019; Ives et al., 2019). Broadly, we contend that coaches, clubs, and governing bodies must strive to re-shape player-environment intentionality with interventions at macro and micro levels. As such, we offer *shaping skilled intentions* (specifically, shaping skilled player-environment intentionality) as an additional principle of Non-linear Pedagogy (see Chow et al., 2016; Woods et al., 2020a for detailed overviews and practical implications) and we continue to discuss practical implications for coaches and coach education in football.

### Markers of Skilled Intentionality in Football

Footballers embody skilled intentions when they deceive and unbalance opponents with fakes and feints. Successful fakes and feints are “markers of skilled intentionality” because they demonstrate a “skillful responsiveness to multiple nesting and nested affordances simultaneously” (van Dijk and Rietveld, 2017 p. 9). These markers are moments that identify skilled intentionality because their success is dependent upon on having an “optimal grip” on the relevant field of affordances (see Bruineberg and Rietveld, 2014; Rietveld and Brouwers, 2017).

In other words, successful fakes and feints mark the moments in which value-directedness facilitates the detection, selection and utilization of nested affordances. In contrast, it is worth considering whether football matches and training sessions devoid of fakes, feints, and deceptive play might be subject to too rigid a value-directedness, and therefore exemplify and or reinforce unskilled intentionality? This may be evident in training sessions whereby players are explicitly told what to do by coaches who communicate abstract and decontextualized knowledge *about* football concepts. As van der Kamp et al. (2019) have outlined, the use of prescriptive pedagogy that conveys knowledge *about* the environment deeply contrasts with (and may inhibit) approaches that educate attention, and direct attunement, toward the environmental properties and local information that theoretically underpins collective behavior and perceptual learning (López-Felip et al., 2020; Woods et al., 2020b).

**FIGURE 1:**
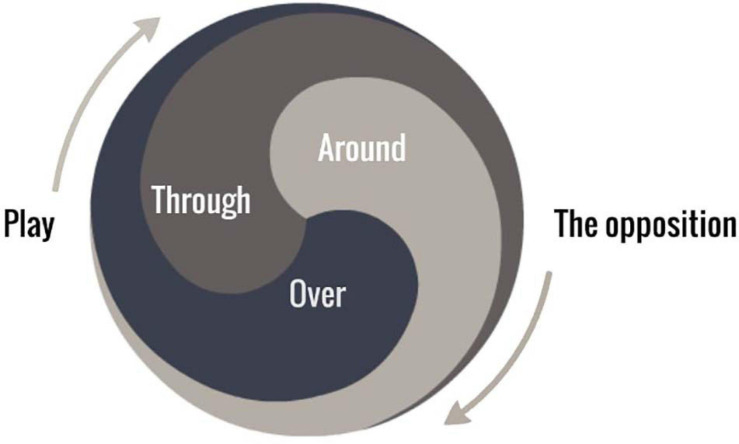
An illustration of the constitutive and nested relation of skilled intentions to play through, around and over the opposition in football.

Here, we propose that unskilled intentionality is evident when teams and players coordinate with a narrow field of relevant affordances in football and team sport. Exemplified by young football players swarming around the ball like “bees around honey” (i.e., a flock of birds) (López-Felip, 2019). We suggest that unskilled intentionality in football might be observed in games, training sessions and even in playing styles that appear one-directional, predictable, and rigidly patterned. In contrast, skilled intentions would always exist in a metastable region of performance (Davids et al., 2008), exhibiting a co-adaptive and constitutive relationship and a tension or balance between doing this or doing that (akin to Yinyang). Illustrated using a football example in Figure 1.

### Shaping Skilled Intentions in Football

To shape the skilled intentions of players, coaches must foster a broad orientation toward informational properties of the football environment. We conceptualize this orientation as a “directedness” (van Dijk and Rietveld, 2017) toward something or someone (Rasmussen et al., 2017), e.g., opponents, teammates, the ball, and subsequent gaps and spaces (i.e., dynamic properties of the football environment). When in possession of the ball, football coaches might foster the skilled intentions (i.e., a directedness) of learners to play *through, around* and *over* the opposition in order to score (see Figure 1). When playing without possession of the ball, the aim becomes stopping the opposition playing through, around or over our team, while simultaneously attempting to win the ball. As opposed to traditional football concepts that analyze and isolate aspects of the game (creating unrelated puzzle pieces), skilled intentions provide synthesis and continuity throughout the game.

Shaping skilled intentions aims to facilitate an optimal level of team coordination and self-organization (i.e., synchrony) without sacrificing opportunities for adaptive, instinctive and potentially creative play. By directing players toward local interactions we avoid the traditional tendency to globally impose tactical plans that aim to control, and often inhibit local interactions (Ribeiro et al., 2019). Patterns of team coordination (i.e., self-organization) that emerge from local interactions are flexible and adaptive to local information, meaning that players can break these patterns in unpredictable ways.

Players who display skilled intentions will balance (i.e., juggling) the nested intentions (seen in Figure 1) moment to moment as they coordinate/synchronize their movements, both on and off the ball, in order to play through, around and over the opposition. Successful fakes and feints on the ball and off the ball embody skilled intentions and can be observed by coaches.

To exemplify shaping intentions, consider a rondo whereby two teammates are defending at the same time (i.e., out of possession) but not together, meaning there is no synchrony or collective coordination to their efforts (demonstrating no attunement to each other’s movements) and the opposition are playing through them too easily. To remedy this situation, a coach can shape intentions by highlighting the balance, or tension, in what the teammates are trying to achieve. In this situation, the initial intention should be to stop the opposition playing *through* (directing attention toward each other’s movements and the gaps/spaces between them), and the nested intention is to simultaneously find ways to win the ball. In the first author’s experience, a coach can successfully shape intentions via skillful session design (size/shape of the playing area, starting positions, and numbers of players) the manipulation of task constraints (points systems, rules, and zones) as well as discussions and direct instruction that helps shape intentions and direct players attention toward key environmental properties. In this case, the movements of *teammates* and the *ball* in relation to the *opponent’s* affordances to play through.

The constant co-adaptation between attack and defense in football (Passos et al., 2016; López-Felip and Turvey, 2017) means that a coordinated defense creates a new challenge for the attack. In this example, more coordinated efforts to stop the opposition playing *through*, simultaneously shines a light on affordances, and concurrently encourages the development of skills required to play *around*. Exemplifying the relations and synthesis is at the core of shaping skilled intentions.

**FIGURE 2:**
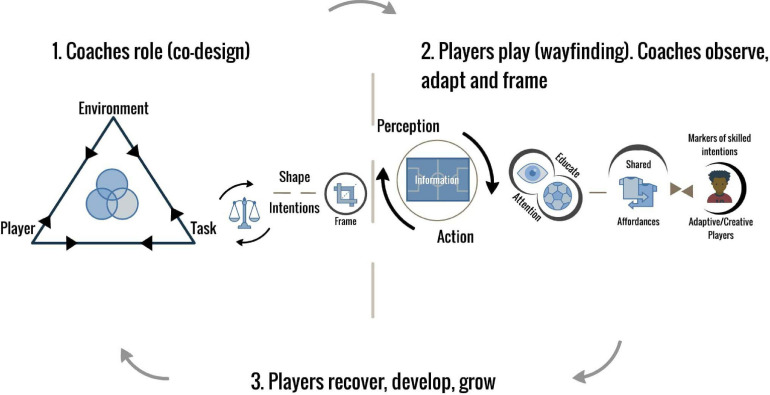
Player learning IN development framework, part of AIK football club’s player development cycle.

At AIK Football Club in Sweden, *shaping skilled intentions* is placed within a broader player development cycle (seen in Figure 2). In this cycle, learning is conceptualized as *wayfinding* (for details see Woods et al., 2020b). The explorative process whereby players find ways to navigate (i.e., overcome challenges) in their football environment via a broad, flexible orientation and value-directedness toward the environmental properties *of* the game, e.g., opponents, teammates, and the ball (line markings, goals etc.).

## Future Research and Applied Considerations

Practitioners must be *aware* of the value-directedness that is *shaping intentions* and the extent to which it may become rigid and inflexible. To gain such an awareness we advocate for the inclusion of ethnographic methods in research and practice; supporting van Dijk and Rietveld (2017) in their argument that ecological psychology would benefit from ethnographic methods that themed the patterned practices of forms of life across multiple grains of analysis.

Here, we acknowledge that ethnographic data predominantly constitutes indirect knowledge *about* the environment, whereas affordances are utilized with knowledge *of*, or direct perception *in*, the environment (van der Kamp et al., 2019). While fully outlining these points is beyond the scope of the current paper it will be important for future research. Crucially, Araújo et al. (2017) stated that gains in direct perception can be mediated through communication *about* the environment and Gibson (1979) explained that “images, pictures, and written-on surfaces afford a special kind of knowledge [*about* the environment] … mediated or indirect, knowledge at second hand” (p. 42; text in brackets added). We propose that second-hand knowledge *about* the environment collected via ethnographic methods can illuminate the value-directedness of player-environment intentionality and play a vital role in aiding coaches’ awareness of value-directedness. However, in the context of learning *in* football, we contend that providing second-hand knowledge *about* the football environment is vastly inferior to facilitating the direct perception of affordances *in* the football environment.

Unfortunately, football coach education, coaching methodology and pedagogy (i.e., instruction/feedback/questioning) seem to focus exclusively on abstract and decontextualized knowledge *about* the football environment (van der Kamp et al., 2019). For example, most football concepts (i.e., switches of play, defensive triangles, inner corridors, etc.) are de-coupled from the dynamic properties *of* the football environment, providing only second-hand representations that do not optimally aid direct perception *in* football. If the aim is learning and optimal performance *in* football then we suggest that communication *about* the football environment avoids the use of conceptual abstractions and representations where possible, and instead utilizes the communication vehicle of shaping skilled intentions.

According to Ribeiro et al. (2019) game models and tactical plans continue to dominate training session design in football coaching. This trend is predicated on the assumption that the self-organization of collective behaviors can be optimally developed by coaches imposing global (abstract and decoupled knowledge *about*) football concepts, from a global-to-local direction, that predict and specify local interactions. As we have argued in this paper, the self-organization of collective behaviors may be optimized by methods that prioritize knowledge *of*, or direct attunement to, the local interactions of environmental properties in football (Ribeiro et al., 2019; López-Felip et al., 2020; Woods et al., 2020b). The arguments in this article support these ideas by suggesting that coaches and coach educators need to prioritize learning through the continuous interactions of players on the pitch and in the game, not through the imposition of verbal instruction of football concepts. We propose that shaping skilled intentions facilitates self-organization in a local to global direction (Ribeiro et al., 2019) and future research is needed to explore this idea in practice and training contexts.

### The Language of Skilled Intentions

For practitioners, it may be useful to distinguish between developing players understanding *about* the game (i.e., improving players ability to talk *about* football using abstract concepts/representations). In contrast to developing players understanding *in* the game (fostering a directedness toward playing through, around and over the opposition) via the perception *of* nested affordances. Future research and applied practice should aim to investigate the extent to which verbal communication (*about* the environment) is useful in enhancing skill development and performance in sport. To aid such an endeavor we offer a distinction between the *language of skilled intentions*: purposefully orientating players toward (i.e., prioritizing coupling to) the properties of the performance environment in order to foster an understanding *in* the game (see Figure 1). Compared with the *language of shared mental models*: coach explanations about abstract concepts, “best decisions,” and representations that may enhance an understanding *about* football, but not necessarily *in* football.

This distinction can be described by referring to the knowledge of a player and a TV commentator. A commentator (or pundit), armed with the latest statistics and data about performance, may display extensive knowledge *about* football performance when presenting a match on TV. Data may be used by the commentator to explain why a certain decision may have been a “better one”: e.g., 60% of shots inside the area are on target and it may have been better to pass not shoot. In contrast, only a player can gain knowledge *of* the football environment, explaining why the player made a specific decision at any one moment. Knowledge *of* the performance environment is gained through experience of informational constraints and perception of affordances while the “ball is rolling” and football players are moving *in* the game, not while players or coaches talk *about* the game.

## Conclusion

In this conceptual analysis and synthesis, we discussed the extent to which social and cultural forces influence the value-directedness of player-environment intentionality and aimed to illustrate that value-directedness can act as a constraint on the skill development of football players. In particular, the value-directedness of intentionality may constitute a sociocultural constraint and limit the affordances that are perceived, and in turn, shape skill development in football. As such, we provided an ecological rationale for the recognition that the values that an individual can express are very much *constrained* by the social, cultural and historical character of the institutions (i.e., commodified football clubs/academies) and the social order (i.e., neoliberal/individualistic forms of life) in which that individual lives.

At an applied level, we remind coaches, practitioners, and sport scientists that training sessions are not blank slates devoid of social, historical, and cultural context/influence. Here, we sought to highlight that sport practitioners need to become more aware of the extent to which sociocultural and historical constraints continuously shape their work (methodologies in practice), and the intentions of players within training sessions and games. Without such an awareness, the social, cultural, and historic constraints of their environment will surreptitiously dominate athlete development, constantly prioritizing solicitations of some affordances over others, subtly influencing skill development, regardless of contemporary developments in motor learning and pedagogical sciences. Our focus in foregrounding skilled intentions *in* football (Figure 1) is not to describe the dynamics of football and or produce more knowledge *about* the game. Rather, it aims to foreshadow knowledge *of* the performance environment to guide coaching practice, enabling coaches to help players to *develop* and *perform* optimally *in* football, directing intention and attention toward knowledge *of* environmental properties *in* the game, fostering skilled intentionality, and co-currently developing exceptional skill. Crucially, shaping skilled intentions offers synthesis and unity to coaching practice by demonstrating that the development of skill and team-coordination (i.e., tactics) are not separate topics requiring different approaches but entirely interdependent aspects of the same whole, deeply related to a relevant field of affordances in football.

## Author Contributions

JV carried out the drafting, conception and design of the manuscript, conceptualized the combination of theoretical, and conceptual frameworks. CM contributed to the conception and design of the manuscript and ensuring that the ideas presented were appropriately investigated and articulated. KD critically revised the manuscript for important intellectual content and ensured that the ideas presented were appropriately investigated and articulated, with particular respect to ecological dynamics. PP revised the manuscript critically for important intellectual content, in particular revising the structure. ML-F reviewed the manuscript for important intellectual content, revised sections articulating concepts from ecological psychology, and football praxis. All authors contributed to the article and approved the submitted version.

## Conflict of Interest

JV was employed as Head of Development (13–19) AIK FF Stockholm and is a co-founder of Player Development Project. ML-F was contracted by Futbol Club Barcelona. The remaining authors declare that the research was conducted in the absence of any commercial or financial relationships that could be construed as a potential conflict of interest.

## Footnotes

1 Path dependency is a system process which demonstrates that once a system is set on a development path, the historically derived (paradigmatic/cultural) modeling (ways of doing science) and emergent structures (reductive/quantitative technology, cultural assumptions) constrain its trajectory of development (Djelic and Quack, 2007).

2 The cross-pollination of different human experiences in society, shaped by samba and capoeira has infused Brazilian football with a preference for a playing style of malandragem (deceptive trickery), yielding the deceptive dribbling skills characterized as “playing with Ginga” (rhythmic body sway) (Uehara et al., 2018).

3 This means that a living system undergoes transitions through states of stability and instabilities as influenced by its intrinsic dynamics (its dispositional tendencies) and external information sources in the environment (Davids, 2015).

4 “Examples of forms of life within the overarching human form of life are builders, English language users, concert-pianists and academics. A human individual typically belongs to multiple partially overlapping forms of life (Rietveld et al., 2018, p. 6).”

5 See here https://www.youtube.com/watch?v=GUPiMFHbbFU

## References

[B1] Aarons E. (2020). *Jürgen Klopp: “Tony Yeboah Had a Big Impact on German Society.” The Guardian.* Available online at: https://www.theguardian.com/football/2020/may/31/jurgen-klopp-tony-yeboah-had-a-big-impact-on-german-society

[B2] Alhadeff-Jones M. (2009). Revisiting Educational Research Through Morin’s Paradigm of Complexity: A Response to Ton Jörg’s Programmatic View. *Complicity Internat. J. Compl. Educ.* 6 61–70.

[B3] Araújo D. (2009). Preface to “ecological approaches to cognition in sport and exercise. *Intern. J. Sport Psychol.* 2009:40.

[B4] Araújo D. Davids K. (2009). Ecological approaches to cognition and action in sport: Ask not what you do, but where you do it. *J. Sport Psychol.* 40 5–37.

[B5] Araújo D. Davids K. (2011). What exactly is acquired during skill acquisition? *J. Consciou. Stud.* 18 7–23.

[B6] Araújo D. Davids K. (2016). Team synergies in sport: Theory and measures. *Front. Psychol.* 7 1–13. 10.3389/fpsyg.2016.01449 27708609 PMC5030782

[B7] Araújo D. Davids K. Hristovski R. (2006). The ecological dynamics of decision making in sport. *Psychol. Sport Exerc.* 7 653–676. 10.1080/1357332052000308792

[B8] Araújo D. Hristovski R. Seifert L. Carvalho J. Davids K. (2017). Ecological cognition: expert decision-making behaviour in sport. *Intern. Rev. Sport Exerc. Psychol.* 9858 1–25. 10.1080/1750984X.2017.1349826

[B9] Baggini J. (2018). *How the world thinks: a global history of philosophy.* London: Granta Books.

[B10] Balagué N. Torrents C. Hristovski R. Kelso J. A. S. (2017). Sport Science Integration. An evolutionary synthesis. *Eur. J. Sport Sci.* 17 51–62. 10.1080/17461391.2016.1198422 27685425

[B11] Bocchi G. Cianci E. Montuori A. Trigona R. Nicolaus O. (2014). Educating for creativity. *World Futures* 70 5–6. 10.1080/02604027.2014.977084

[B12] Bowes I. Jones R. L. (2006). Working at the Edge of Chaos: Understanding Coaching as a Complex. *Sport PSychol.* 20 235–245. 10.1123/tsp.20.2.235

[B13] Bruineberg J. Rietveld E. (2014). Self-organization, free energy minimization, and optimal grip on a field of affordances. *Front. Hum. Neurosci.* 8 1–14. 10.3389/fnhum.2014.00599 25161615 PMC4130179

[B14] Bundesliga (2013). *Okocha’s Magic Dancing Dribble.* https://www.youtube.com/watch?v=GUPiMFHbbFU (accessed date September 27, 2013).

[B15] Button C. Seifert L. Chow J.-Y. Araújo D. Davids K. (2020). *Dynamics of Skill Acquisition: An Ecological Dynamics rationale*, 2nd Edn. Champaign: Human Kinetics.

[B16] Chow J. Y. Davids K. Button C. Renshaw I. (2016). *Nonlinear pedagogy in skill acquisition: an introduction.* England: Routledge.

[B17] Chow J.-Y. Davids K. Shuttleworth R. Araújo D. (2020). “Ecological dynamics and transfer from practice to performance in sport,” in *Skill Acquisition in Sport: Research, Theory and Practice*, 3rd Edn, eds Williams A. M. Hodges N. (London: Routledge), 330–344. 10.4324/9781351189750-18

[B18] Coutinho P. Mesquita I. Davids K. Fonseca A. M. Côté J. (2016). How structured and unstructured sport activities aid the development of expertise in volleyball players. *Psychol. Sport Exerc.* 25 51–59. 10.1016/j.psychsport.2016.04.004

[B19] Curran T. Hill A. P. (2019). Perfectionism is increasing over time: A meta-analysis of birth cohort differences from 1989 to 2016. *Psychol. Bull.* 145 410–429. 10.1037/bul0000138 29283599

[B20] Davids K. (2015). Athletes and sports teams as complex adaptive system: a review of implications for learning design. *Rev. Int. Ciencias Deporte* 11 226–244. 10.5232/ricyde2015.03904

[B21] Davids K. Araújo D. (2010). The concept of “Organismic Asymmetry” in sport science. *J. Sci. Med. Sport* 13 633–640. 10.1016/j.jsams.2010.05.002 20580313

[B22] Davids K. Button C. Bennett S. J. (2008). Dynamics of skill acquisition. *Human Kinet.* 3:1.

[B23] Davids K. Handford C. Williams M. (1994). The natural physical alternative to cognitive theories of motor behaviour: an invitation for interdisciplinary research in sports science? *J. Sports Sci.* 12:6. 10.1080/02640419408732202 7853448

[B24] Denison J. Jones L. Mills J. P. (2019). Becoming a good enough coach. *Sports Coach. Rev.* 8:1435361. 10.1080/21640629.2018.1435361

[B25] Djelic M. L. Quack S. (2007). Overcoming path dependency: Path generation in open systems. *Theory Soc.* 36 161–186. 10.1007/s11186-007-9026-0

[B26] Dunwoody P. T. (2006). The neglect of the environment by cognitive psychology. *J. Theor. Philosop. Psychol.* 26 139–153. 10.1037/h0091271

[B27] Fajen B. R. Riley M. A. Turvey M. T. (2009). Information, affordances, and the control of action in sport. *Internat. J. Sport Psychol.* 40 79–107.

[B28] Gale L. A. Ives B. A. Potrac P. A. Nelson L. J. (2019). Trust and Distrust in Community Sports Work: Tales From the “Shop Floor”. *Soc. Sport J.* 36:3. 10.1123/ssj.2018-0156

[B29] Gibson E. J. (1988). Exploratory behaviour in the development of perceiving, acting, anfd the acuiring of knowledge. *Ann. Rev. Psychol.* 39 1–41. 10.1146/annurev.ps.39.020188.000245

[B30] Gibson J. J. (1966). *The senses considered as perceptual systems.* Boston, MA: Houghton Mifflin.

[B31] Gibsons J. J. (1979). *The ecological approach to visual perception.* Boston, MA: Houghton Mifflin.

[B32] Henriksen K. Stambulova N. (2017). “Creating optimal environments for talent development: A holistic ecological approach,” in *Routledge Handbook of Talent Identification and Development in Sport*, eds Baker J. Cobley S. Schorer J. Wattie N. (England: Routledge), 271–284.

[B33] Hodges B. H. Baron R. M. (1992). Values as constraints on affordances: Perceiving and acting properly. *J. Theor. Soc. Behav.* 22 263–294. 10.1111/j.1468-5914.1992.tb00220.x

[B34] Ives B. A. Gale L. A. Potrac P. A. Nelson L. J. (2019). Uncertainty, shame and consumption: negotiating occupational and non-work identities in community sports coaching. *Sport Educ. Soc.* 0 1–17. 10.1080/13573322.2019.1699522

[B35] Jones R. L. Potrac P. Cushion C. Ronglan L. T. (2010). *The sociology of sports coaching.* England: Routledge, 10.4324/9780203865545

[B36] Lobo L. Heras-Escribano M. Travieso D. (2018). The history and philosophy of ecological psychology. *Front. Psychol.* 9:02228. 10.3389/fpsyg.2018.02228 30555368 PMC6280920

[B37] López-Felip M. A. (2019). *Collective Behavior in Dissipative Systems: Flocking and Futbol.* Doctoral Dissertations, Storrs, CT: University of Connecticut, 2316.

[B38] López-Felip M. A. Turvey M. T. (2017). Desideratum for GUT: A functional semantics for sport. *Hum. Move. Sci.* 56 169–172. 10.1016/j.humov.2017.05.002 28501422

[B39] López-Felip M. A. Pettersen M. N. Harrison H. S. Dixon J. A. (2020). *Ball positioning as key to understand collective behavior. In Football Analytics: 2021. The role of context in transferring analytics to the pitch.* Barcelona: Barça Innovation Hub, 122–139.

[B40] O’ Sullivan M. Woods C. T. Vaughan J. Davids K. (2021). Towards a contemporary player learning in development framework for sports practitioners. *Internat. J. Sports Sci. Coach.* 2021:2335. 10.1177/17479541211002335

[B41] Passos P. Araújo D. Davids K. (2016). Competitiveness and the process of co-adaptation in team sport performance. *Front. Psychol.* 7:1562. 10.3389/fpsyg.2016.01562 27777565 PMC5056172

[B42] Potrac P. Jones R. L. Gilbourne D. Nelson L. (2013). Handshakes, BBQs, and bullets’: self-interest, shame and regret in football coaching. *Sports Coach. Rev.* 1 79–92. 10.1080/21640629.2013.768418

[B43] Potrac, Paul, Mallett C. Greenough K. Nelson L. (2017). Passion and paranoia: an embodied tale of emotion, identity, and pathos in sports coaching. *Sports Coach. Rev.* 6:2. 10.1080/21640629.2017.1367067

[B44] Raja V. (2019). From metaphor to theory: the role of resonance in perceptual learning. *Adapt. Behav.* 27:6. 10.1177/1059712319854350

[B45] Rasmussen L. J. T. Østergaard L. D. Glãveanu V. P. (2017). Creativity as a developmental resource in sport training activities. *Sport Educ. Soc.* 3322 1–16. 10.1080/13573322.2017.1403895

[B46] Reed E. S. (1988). *James J. Gibson and the psychology of perception.* New Haven: Yale University Press.

[B47] Renshaw I. Chow J. Y. (2019). A constraint-led approach to sport and physical education pedagogy. *Phys. Educ. Sport Pedag.* 24:2. 10.1080/17408989.2018.1552676

[B48] Renshaw I. Davids K. Newcombe D. Roberts W. (2019). *The Constraints-led Approach: Principles for Sports Coaching and Practice Design.* Abingdon: Routledge.

[B49] Renshaw I. Araújo D. Button C. Chow J. Y. Davids K. Moy B. (2016). Why the Constraints-Led Approach is not Teaching Games for Understanding: a clarification. *Phys. Educ. Sport Pedag.* 21:5. 10.1080/17408989.2015.1095870

[B50] Ribeiro J. Davids K. Araújo D. Guilherme J. Silva P. Garganta J. (2019). Exploiting Bi-Directional Self-Organizing Tendencies in Team Sports: The Role of the Game Model and Tactical Principles of Play. *Front. Psychol.* 10:2213. 10.3389/fpsyg.2019.02213 31649579 PMC6794429

[B51] Rietveld E. Brouwers A. A. (2017). Optimal grip on affordances in architectural design practices: an ethnography. *Phenomenol. Cogn. Sci.* 16 545–564. 10.1007/s11097-016-9475-x

[B52] Rietveld E. Denys D. van Westen M. (2018). *Ecological-Enactive Cognition as Engaging with a Field of Relevant Affordances: The Skilled Intentionality Framework (SIF).* Oxford: Oxford University Press.

[B53] Roderick M. (2006). *The Work of Professional Football.* New York, NY: Taylor & Francis.

[B54] Rossing N. N. Skrubbeltrang L. S. (2016). The language of football: a cultural analysis of selected world cup nations. *Sport Soc.* 0437 1–13. 10.1080/17430437.2016.1158478

[B55] Rothwell M. D. K. Stone J. A. Sullivan M. O. Vaughan J. Newcombe D. J. Shuttleworth R. (2020). A Department of Methodology Can Coordinate Transdisciplinary Sport Science Support. *J. Exp.* 3:1.

[B56] Rothwell M. Davids K. Stone J. (2018). Harnessing Socio-cultural Constraints on Athlete Development to Create a Form of Life. *J. Exp.* 2018:2018.

[B57] Rothwell M. Stone J. Davids K. (2019). Exploring Forms of Life in Player Development Pathways: The Case of British Rugby League. *J. Motor Learn. Dev.* 7 242–260. 10.1123/jmld.2018-0020

[B58] Runeson S. Juslin P. Olsson H. (2000). Visual perception of dynamic properties: Cue heuristics versus direct-perceptual competence. *Psycholog. Rev.* 107:3. 10.1037/0033-295X.107.3.525 10941279

[B59] Stambulova N. B. Ryba T. V. (2014). A critical review of career research and assistance through the cultural lens: towards cultural praxis of athletes’ careers. *Internat. Rev. Sport Exer. Psychol.* 7:1. 10.1080/1750984X.2013.851727

[B60] Teques P. Araújo D. Seifert L. del Campo V. L. Davids K. (2017). The resonant system: Linking brain–body–environment in sport performance? *Prog. Brain Res.* 2017:234. 10.1016/bs.pbr.2017.06.001 29031470

[B61] Turvey M. T. (1990). Coordination. *Am. Psychol.* 45:938. 10.1037//0003-066x.45.8.938 2221565

[B62] Turvey M. T. Shaw R. E. Reed E. S. Mace W. M. (1981). Ecological laws of perceiving and acting: In reply to Fodor and Pylyshyn (1981). *Cognition* 9:90002. 10.1016/0010-0277(81)90002-0 7197604

[B63] Uehara L. Button C. Araújo D. Renshaw I. Davids K. Falcous M. (2018). The Role of Informal, Unstructured Practice in Developing Football Expertise: The Case of Brazilian Pelada. *J. Exp.* 1:3.

[B64] Uehara L. Button C. Falcous M. Davids K. (2016). Contextualised skill acquisition research: a new framework to study the development of sport expertise. *Phys. Educ. Sport Pedag.* 21:2. 10.1080/17408989.2014.924495

[B65] van der Kamp J. Withagen R. Orth D. (2019). On the Education About/of Radical Embodied Cognition. *Front. Psychol.* 10 1–9. 10.3389/fpsyg.2019.02378 31749732 PMC6848271

[B66] van Dijk L. Rietveld E. (2017). Foregrounding sociomaterial practice in our understanding of affordances: The skilled intentionality framework. *Front. Psychol.* 7 1–12. 10.3389/fpsyg.2016.01969 28119638 PMC5220071

[B67] Vaughan J. (2014). *Developing creative football players: A psychological needs perspective.* St Lucia: The University of Queensland, 10.14264/uql.2015.145 MPhil Thesis.

[B68] Vaughan J. (2020). *Creativity in football: Conceptual frameworks and cultural case studies to inform coaching praxis.* Ph.D. Thesis, Brisbane: The University of Queensland 10.14264/uql.2020.87.

[B69] Vaughan J. Mallett C. J. Davids K. Potrac P. López-Felip M. A. (2019). Developing Creativity to Enhance Human Potential in Sport: A Wicked Transdisciplinary Challenge. *Front. Psychol.* 10:2090. 10.3389/fpsyg.2019.02090 31572271 PMC6753247

[B70] Withagen R. Araújo D. de Poel H. J. (2017). Inviting affordances and agency. *N. Ideas Psychol.* 45:002. 10.1016/j.newideapsych.2016.12.002

[B71] Wittgenstein L. (1953). *Philosophical Investigations.* Oxford: Blackwell.

[B72] Woods C. T. McKeown I. Rothwell M. Araújo D. Robertson S. Davids K. (2020a). Sport Practitioners as Sport Ecology Designers: How Ecological Dynamics Has Progressively Changed Perceptions of Skill “Acquisition” in the Sporting Habitat. *Front. Psychol.* 11:1–15. 10.3389/fpsyg.2020.00654 32390904 PMC7194200

[B73] Woods C. T. Rudd J. Robertson S. Davids K. (2020b). Wayfinding: How Ecological Perspectives of Navigating Dynamic Environments Can Enrich Our Understanding of the Learner and the Learning Process in Sport. *Sports Med. Open.* 6:51. 10.1186/s40798-020-00280-9 33113029 PMC7593371

